# A little ER stress isn’t bad: the IRE1**α**/XBP1 pathway shapes ILC3 functions during intestinal inflammation

**DOI:** 10.1172/JCI182204

**Published:** 2024-07-01

**Authors:** Cinzia Fionda, Giuseppe Sciumè

**Affiliations:** 1 Department of Molecular Medicine, Sapienza University of Rome, Rome, Italy.; 2 Laboratory affiliated to Istituto Pasteur Italia – Fondazione Cenci Bolognetti, Rome, Italy.

## Abstract

Type 3 innate lymphoid cells (ILC3s) are key regulators of intestinal homeostasis and epithelial barrier integrity. In this issue of the *JCI*, Cao and colleagues found that a sensor of endoplasmic reticulum (ER) stress, the inositol-requiring kinase 1α/X-box–binding protein 1 (IRE1α/XBP1) pathway, fine-tuned the functions of ILC3s. Activation of IRE1α and XBP1 in ILC3s limited intestinal inflammation in mice and correlated with the efficacy of ustekinumab, an IL-12/IL-23 blocker, in patients with Crohn’s disease. These results advance our understanding in the use of ILCs as biomarkers not only to predict disease outcomes but also to indicate the response to biologicals in patients with inflammatory bowel disease.

## The IRE1α/XBP1 pathway prevents unresolved ER stress in ILC3s

Innate immune cells ensure organismal homeostatic functions and immune surveillance by rapidly triggering specialized effector functions in response to changes in the microenvironment. Fast responses, however, require extensive protein synthesis, folding, and trafficking, all functions regulated by the endoplasmic reticulum (ER). Cells that are not able to accommodate these needs undergo accumulation of unfolded or misfolded proteins, ER stress, and activation of distinct phylogenetically conserved signaling pathways falling under the umbrella of the unfolded protein response (UPR) (1). Inositol-requiring enzyme 1α (IRE1α) is one of the three major initiators of the UPR and has the primary function of restoring ER homeostasis or inducing programmed cell death when the overall ER status is compromised. In immune cells, physiological and pathological activation of the UPR can affect not only survival but also effector functions and cell fate decisions (2). In this issue of the *JCI*, Cao and colleagues identified a connection between the UPR and the function of type 3 innate lymphoid cells (ILC3s) (3). These cells play crucial roles in maintaining intestinal homeostasis and barrier functions and are defined by expression of the signature cytokines IL-22 and IL-17 and the transcription factor Rorγt (4, 5). Cao et al. (3) found that intestinal ILC3s express high levels of the *ERN1* transcript, which encodes IRE1α, the canonical transmembrane sensor of the UPR. When activated, IRE1α functions as an endoribonuclease cleaving X-box protein 1 (*XBP1*) mRNA. This process leads to generation of a spliced mRNA isoform that is translated to the active transcription factor, namely XBP1s. In turn, XBP1s regulates the expression of numerous UPR genes, including ER chaperones, oxido-reductases, and other proteins involved in the adaptation to ER stress (1). Cao and co-authors determined distinct regulatory mechanisms underlying IRE1α/XBP1 activation in ILC3s and revealed that conditional deletion of *Ern1* in Rorγt-expressing cells (using *Ire1α^ΔRorc^* mice) led to unresolved ER stress and increased expression of proapoptotic genes in ILC3s during inflammation (3).

## Regulation of IRE1α and XBP1 activation in ILC3s

**Figure 1:**
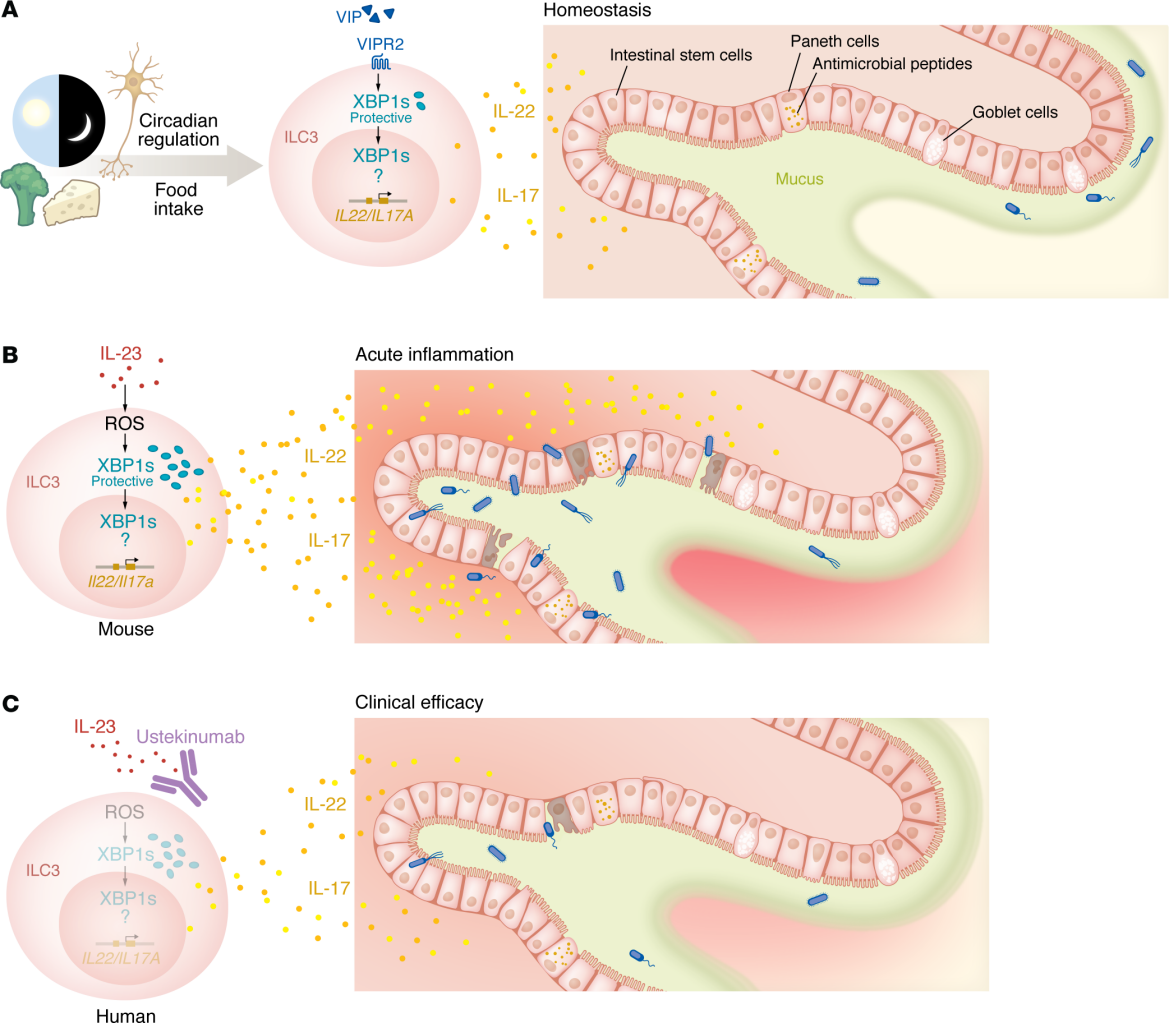
XBP1s has a role in controlling IL-22 production from intestinal ILC3s. (**A**) XBP1s expression in ILC3s follows a circadian rhythm and can be induced by VIP, a neuropeptide produced by enteric neurons during feeding. XBP1s triggers IL-22 release, which helps maintain epithelial barrier integrity. (**B**) Inflammatory cytokines, namely IL-23 and IL-1β, induce mitochondrial ROS–mediated IRE1α/XBP1 pathway activation. XBP1s is required for optimal IL-22 and IL-17 production by ILC3s and protects these cells from unresolved ER stress. Moreover, XBP1s in ILC3s is required for protection against *C. rodentium* and *C. difficile* and to avoid aberrant acute intestinal inflammation in mice. (**C**) The expression levels of XBP1s in ILC3s in intestinal mucosa correlate with the response to the therapeutic antagonist anti–IL-12/IL-23 antibody (also known as ustekinumab) in patients with IBD.

Cao et al. (3) reported that expression of XBP1s in ILC3s follows a robust circadian rhythm and can be induced by the vasoactive intestinal peptide (VIP), a neuropeptide produced by enteric neurons during feeding (Figure 1A). While the role of feeding-dependent activation of VIP in ILC3s is still debated, light exposure heavily synchronizes the functions of ILC3s in the intestine (6). Thus, one could speculate that circadian regulation of IRE1α/XBP1 in ILC3s has the potential to work as a cytoprotector during “rush” hours as well as prior to food intake. A similar mechanism of action can be postulated for ILC2s, since these cells are also tightly regulated by circadian rhythm and VIP (6) and express even higher levels of *Ern1* than do ILC3s (3). The importance of rhythmic regulation in the ER stress response is also underlined by the function of XBP1s, which serves as a transcriptional regulator controlling the cell-autonomous 12-hour clock (7). Although the authors did not observe gross homeostatic alterations in the intestine of *Ire1α^ΔRorc^* mice (3), the findings discussed above warrant further studies aimed at dissecting the role of central and cell-autonomous regulation of the UPR in ILC-mediated immunity. Two key aspects highlighted by Cao and co-authors included (a) the requirement of IRE1α/XBP1 for mouse and human ILC3s to reach the optimal secretory potential of IL-22 and IL-17 upon IL-23 and IL-1β stimulation, and (b) the ability of mitochondrial ROS to selectively activate the IRE1α/XBP1 pathway among the three UPR branches (3). While cytokine-mediated activation of IRE1α and XBP1s has been previously shown to regulate NK cell proliferation (8), it remains to be determined whether the selective activation of IRE1α/XBP1 mediated by ROS and/or XBP1s-dependent cytokine production are generalizable to other ILCs. Thus, XBP1s establishes a positive feedback mechanism that has the potential to amplify the secretory functions of ILC3s, at multiple levels, such as by regulating the fitness and/or production of cytokines (3). This function is well described in B cells, which upregulate the UPR upon activation and differentiation into plasma cells for optimal antibody secretion (9). Since members of the UPR can bind to the promoter of cytokines such as IL-6 or IL-23 in other innate immune cells (10, 11), these observations are open to question as to whether the expression of cytokines can also be tuned by direct regulation of XBP1s in ILC3s. This topic might be tackled in future studies by establishing whole-genome maps of XBP1s-DNA binding in these cells.

## XBP1s and ILC3s in intestinal inflammation

Chronic ER stress and aberrant UPR signaling are recognized as pathological features of many diseases, including metabolic disorders, inflammation, neuropathologies, microbial infections, and cancer. Although the mechanisms linking ER stress and these conditions are still puzzling, activation of apoptosis and promotion of inflammation are two key driving factors (12). ER stress and SNPs in the *XBP1* locus have also been linked with the risk of inflammatory bowel disease (IBD) development in humans (13). The UPR is particularly active on gut epithelial cells with robust secretory properties, namely Paneth and goblet cells. Thus, it is not surprising that XBP1 deficiency in intestinal epithelial cells results in spontaneous enteritis and increased susceptibility to experimental colitis, characterized by exaggerated ER stress, apoptotic Paneth cell loss, reduction of goblet cells, impaired antimicrobial function, and inflammation (13). Using distinct genetic approaches, Cao and colleagues demonstrate that the functional impairment of *Ern1*-deficient ILC3s was sufficient to cause more severe dextran sulfate sodium–induced (DSS-induced) colitis and high vulnerability to *Citrobacter rodentium* and *Clostridium difficile* infections (3). The effects on inflammation and disruption of intestinal barrier integrity in *Ire1α^ΔRorc^* mice could be highly mitigated by exogenous administration of IL-22. These findings are in line with the results obtained using *Il23r^–/–^ Rag2^–/–^* mice, which showed that the innate IL-23/IL-22 axis is protective in conditions of acute colitis (14). However, during intestinal inflammation, the role of these cytokines in ILC3s is context and insult dependent, and it cannot solely be considered beneficial. Accordingly, ILC3s have deleterious effects in mouse models of chronic inflammation due to the pathogenic functions of sustained IL-23 stimulation (15–17). These observations can explain why the frequency of XBP1s^+^ ILC3s in intestinal mucosa correlates with the response to therapeutic anti–IL-12/IL-23 antibody treatment (Figure 1B), as defined by Cao and colleagues, as well as why these cells could also be considered pathogenic in patients with IBD (4, 5).

## ILC3s as biomarkers in patients with IBD

Overall, while the study by Cao et al. reveals a fundamental role for the IRE1α/XBP1 pathway in driving IL-23–dependent ILC3 responses (3), it also generates several interesting questions. What is the relationship among the circadian rhythm, the UPR, and intestinal inflammation? Can disruption of the homeostatic functions of ILC3s alter their responses during colitis? Can these findings be generalized to other ILCs or to other tissues and diseases? What is certain is that, being highly sensitive to changes in the intestinal microenvironment, ILC3s can offer a still underexploited tool for the rapid tracking of modifications of the cytokine milieu, providing a snapshot of the active drivers of tissue inflammation. This information can lead to optimized treatments and a better choice of biologicals for patients. Since there is an urgent need for effective biomarkers for the diagnosis, prognosis, and treatment of IBD, the identification of XBP1s^+^ ILC3s as a possible prognostic factor has important translational implications. However, larger, independent cohorts of patients with IBD will need to be tested. Similarly, evaluation of integrative interaction networks in individuals carrying SNPs in the *XBP1* locus will help to better delineate the contribution of XBP1s in IBD and, hopefully, to identify additional molecular mediators involved in the complex regulation of intestinal homeostasis and inflammation. Finally, comprehensive single-cell RNA-Seq of biopsies from patients with Crohn’s disease and ulcerative colitis and the rapid advances of spatial transcriptomics and proteomics approaches will allow the identification of biomarkers and the deconvolution of interactions among immune cells, stromal cells, neurons, and epithelial cells.

## Version 1 07/01/2024

Electronic publication

## References

[1] Walter P, Ron D (2011). The unfolded protein response: from stress pathway to homeostatic regulation. Science.

[2] Di Conza G (2023). Control of immune cell function by the unfolded protein response. Nat Rev Immunol.

[3] Cao S (2024). The IRE1α/XBP1 pathway sustains cytokine responses of group 3 innate lymphoid cells in inflammatory bowel disease. J Clin Invest.

[4] Horn V, Sonnenberg GF Group 3 innate lymphoid cells in intestinal health and disease. Nat Rev Gastroenterol Hepatol.

[5] Forkel M, Mjösberg J (2016). Dysregulation of group 3 innate lymphoid cells in the pathogenesis of inflammatory bowel disease. Curr Allergy Asthma Rep.

[6] Wang Q, Colonna M (2020). Keeping time in group 3 innate lymphoid cells. Nat Rev Immunol.

[7] Zhu B (2017). A cell-autonomous mammalian 12 hr clock coordinates metabolic and stress rhythms. Cell Metab.

[8] Dong H (2019). The IRE1 endoplasmic reticulum stress sensor activates natural killer cell immunity in part by regulating c-Myc. Nat Immunol.

[9] Van Anken E (2021). Molecular evaluation of endoplasmic reticulum homeostasis meets humoral immunity. Trends Cell Biol.

[10] Martinon F (2010). TLR activation of the transcription factor XBP1 regulates innate immune responses in macrophages. Nat Immunol.

[11] Goodall JC (2010). Endoplasmic reticulum stress-induced transcription factor, CHOP, is crucial for dendritic cell IL-23 expression. Proc Natl Acad Sci U S A.

[12] Marciniak SJ (2022). Pharmacological targeting of endoplasmic reticulum stress in disease. Nat Rev Drug Discov.

[13] Kaser A (2008). XBP1 links ER stress to intestinal inflammation and confers genetic risk for human inflammatory bowel disease. Cell.

[14] Cox JH (2012). Opposing consequences of IL-23 signaling mediated by innate and adaptive cells in chemically induced colitis in mice. Mucosal Immunol.

[15] Buonocore S (2010). Innate lymphoid cells drive interleukin-23-dependent innate intestinal pathology. Nature.

[16] Chen L (2015). IL-23 activates innate lymphoid cells to promote neonatal intestinal pathology. Mucosal Immunol.

[17] Paustian AMS (2017). Continuous IL-23 stimulation drives ILC3 depletion in the upper GI tract and, in combination with TNFα, induces robust activation and a phenotypic switch of ILC3. PLoS One.

